# Supplementary figures and images for: Effect of Compound Kushen Injection, a Natural Compound Mixture, and Its Identified Chemical Components on Migration and Invasion of Colon, Brain, and Breast Cancer Cell Lines
Source: Front Oncol. 2019 Apr 26;9:314. doi: 10.3389/fonc.2019.00314 (PMC6498862; doi:10.3389/fonc.2019.00314)

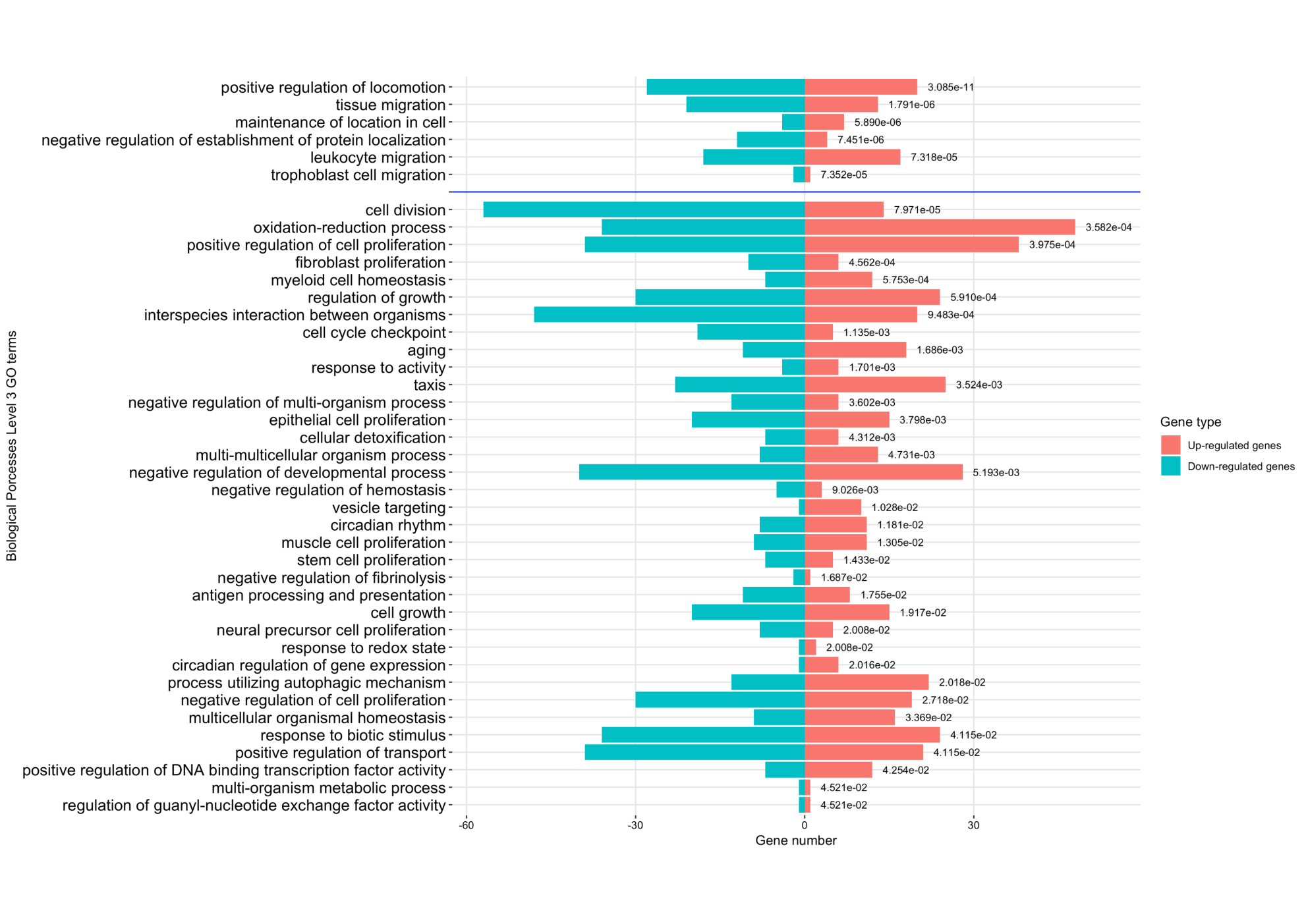

Supplement: Supplementary Image 1 — Functional classification of genes by GO over-representation analyses. Over-represented GO terms (Biological Process, BP = 3) for differentially expressed (DE) genes were identified from MDA-MB-231 cells treated by CKI. Upregulated and downregulated genes contained in each term were shown in red and green, respectively. GO terms shown above the blue line were significant terms related to migration. [file Image_1.TIF]

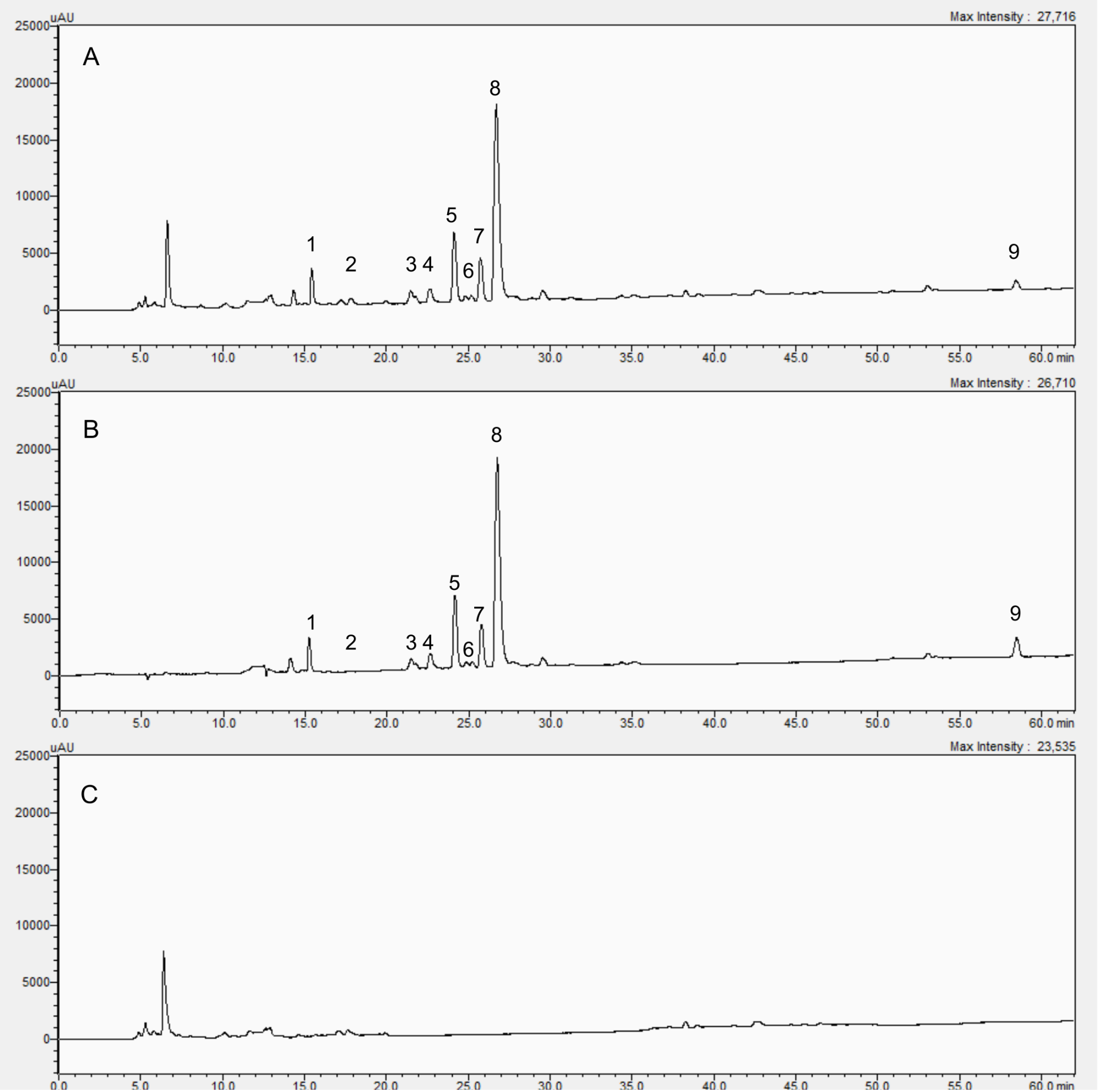

Supplement: Supplementary Image 2 — HPLC profiles of the components present in (A) CKI, (B) MJ, and (C) MN fractions. Samples (50 μl at 1 mg/ml) were run through a C18 semi-preparative column. Numbers indicate the nine major compounds; 1: macrozamin, 2: adenine, 3: n-methylcytisine, 4: sophoridine, 5: matrine, 6: sophocarpine, 7: oxysophocarpine, 8: oxymatrine, and 9: trifolirhizin. [file Image_2.TIF]

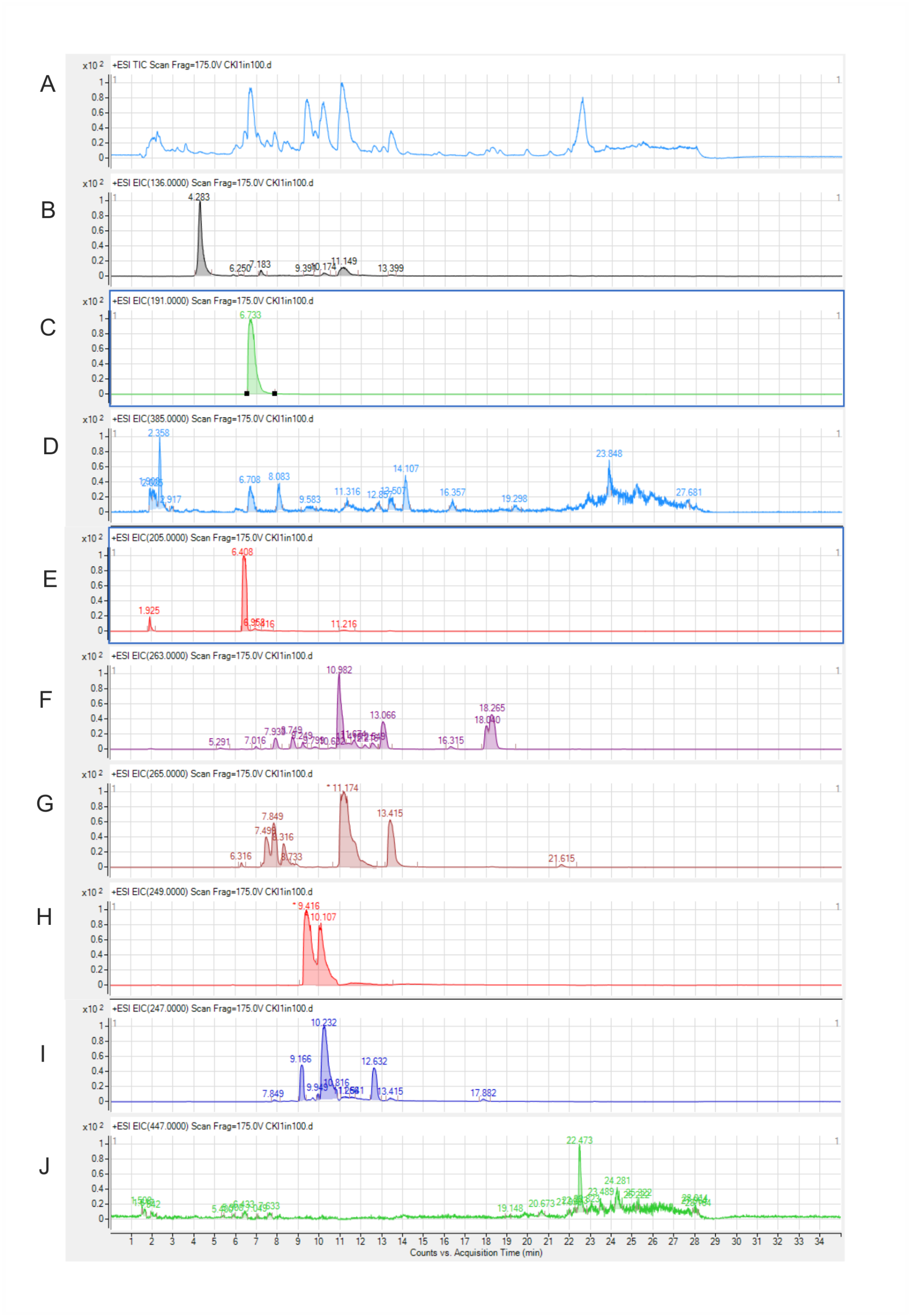

Supplement: Supplementary Image 3 — (A) Total ion chromatogram (TIC) for CKI in 1 in 100 dilution from 25 mg/ml of stock concentration. Single peaks were extracted based on the molecular mass. (B) cytisine (spike in control), (C) macrozamin, (D) adenine, (E) n-methylcytisine, (F) sophoridine and matrine (similar molecular mass with different retention time) (G) oxysophocarpine, (H) oxymatrine, (I) sophocarpine, and (J) trifolirhizin. [file Image_3.TIF]

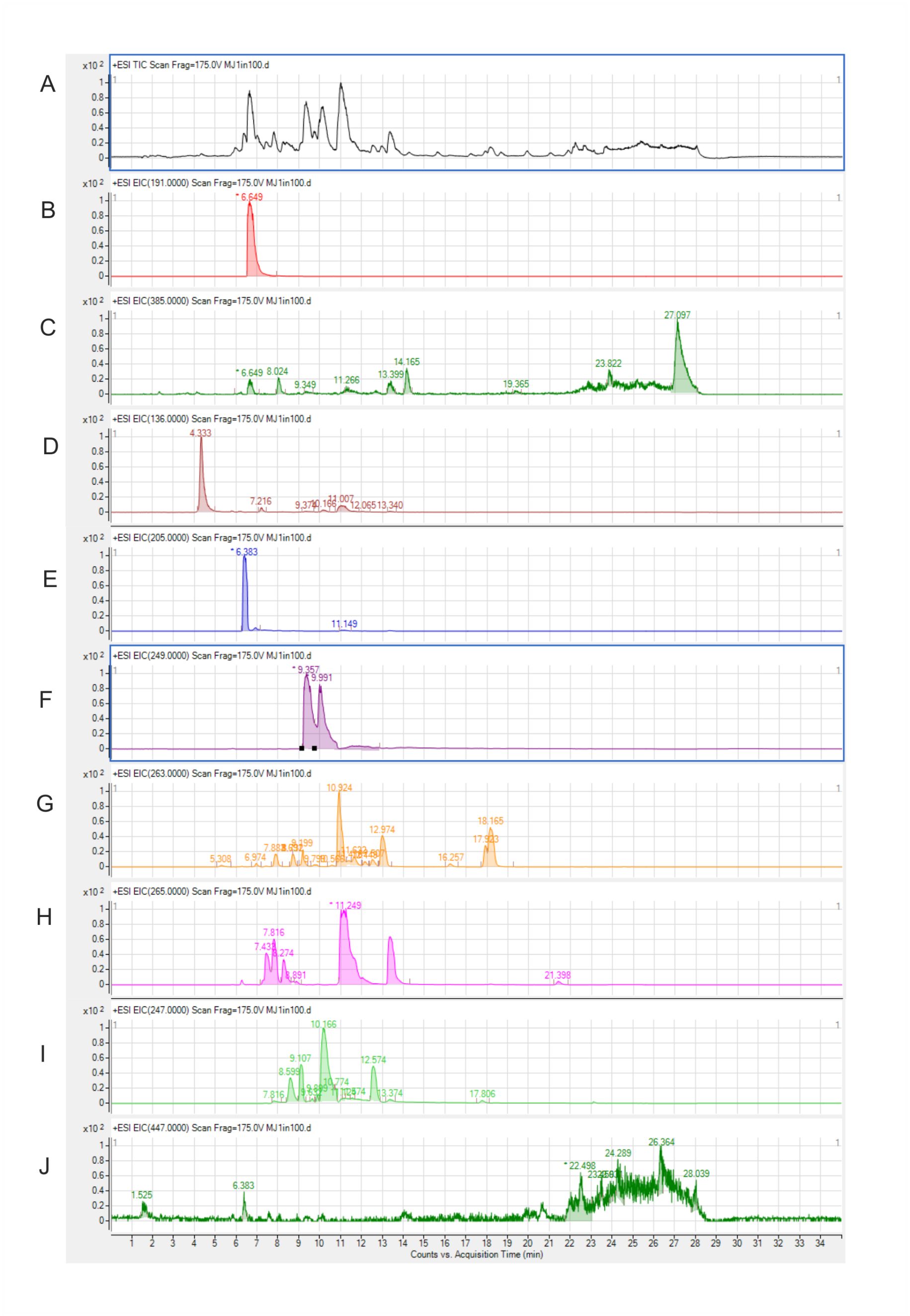

Supplement: Supplementary Image 4 — (A) Total ion chromatogram (TIC) for MJ in 1 in 100 dilution from 25 mg/ml of stock concentration. Single peaks were extracted based on the molecular mass. (B) adenine, (C) cytisine (spike in control), (D) macrozamin, (E) n-methylcytisine, (F) sophoridine, and matrine (similar molecular mass with different retention time) (G) oxysophocarpine, (H) oxymatrine, (I) sophocarpine, and (J) trifolirhizin. [file Image_4.TIF]

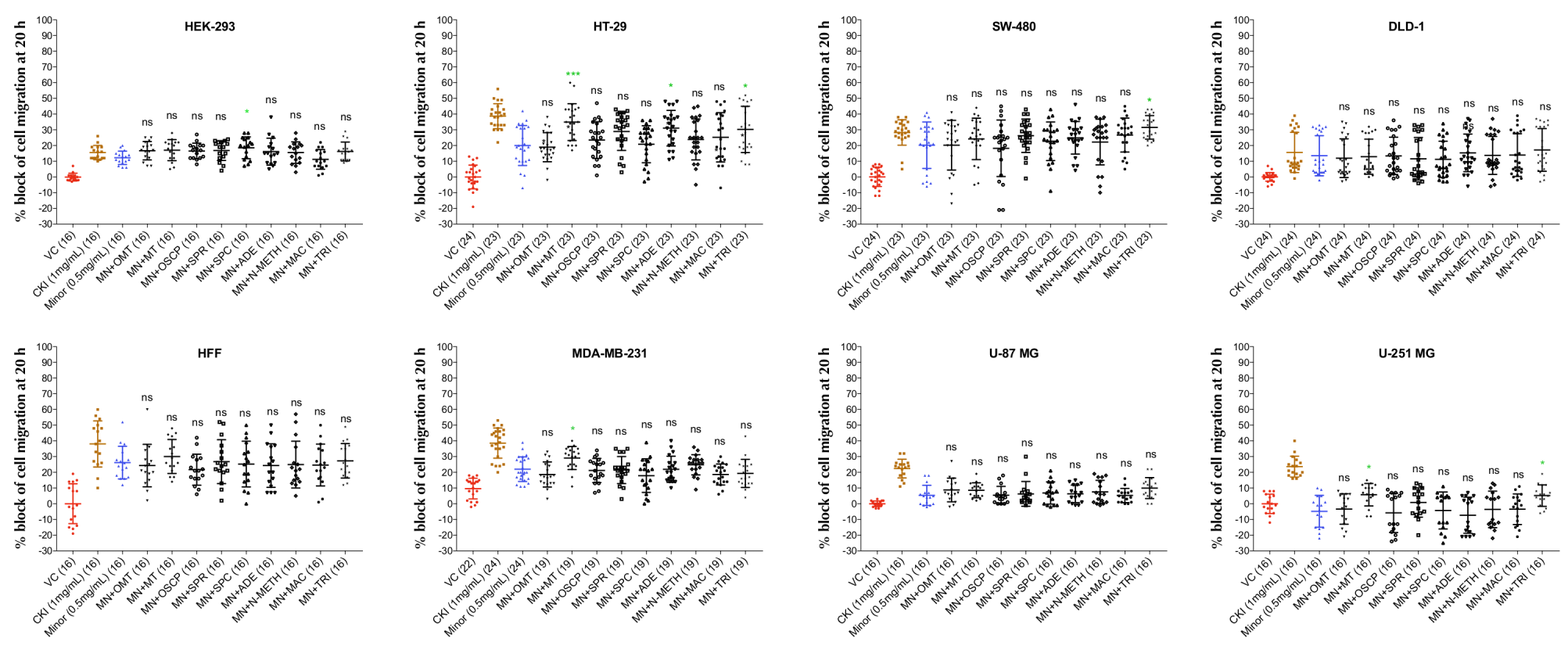

Supplement: Supplementary Image 5 — Combinatorial analysis of the effects of MN with each of the nine major individual compounds, analyzed in eight cell lines with wound closure assays. Data were normalized to results with 0.5 mg/ml minor (MN) alone. Significantly increased or decreased percent block of migration resulting from the addition of major compounds is shown as *p < 0.05, **p < 0.01, ***p < 0.001, and not significant (ns). Data are mean ± SD. [file Image_5.TIF]
