# Supplementary material for: Effect of Compound Kushen Injection, a Natural Compound Mixture, and Its Identified Chemical Components on Migration and Invasion of Colon, Brain, and Breast Cancer Cell Lines
Source: Front Oncol. 2019 Apr 26;9:314. doi: 10.3389/fonc.2019.00314 (PMC6498862; doi:10.3389/fonc.2019.00314)
Supplement: Supplementary Table 2 — Concentrations of Matrigel and number of cells used for each cell line in transwell invasion assay. [file Table_2.DOCX]

| Cell line | Number of cells/ml | Concentration of matrigel (Stock: 10 mg/ml) | Matrigel loading Volume | Incubation time (hours) |
| --- | --- | --- | --- | --- |
| MDA-MB-231 | 1X 10^6^ | 1 in 40 | 40 µl | 5 |
| U-87 | 5X 10^5^ | 1 in 30 | 40 µl | 4-5 |
| U-251 | 5X 10^5^ | 1 in 40 | 40 µl | 4-5 |
| DLD-1 | 1X 10^6^ | 1 in 100 | 40 µl | 24 |
| SW-480 | 1.5X 10^6^ | 1 in 200 | 40 µl | 24 |
| HT-29 | 2.5X 10^6^ | 1 in 400 | 40 µl | 24 |
| HEK-293 | 5X 10^5^ | 1 in 40 | 40 µl | 24 |
| HFF | 5X 10^5^ | 1 in 150 | 40 µl | 24 |

Supplementary Table 2: Concentration of Matrigel and number of cells used for each cell line in transwell invasion assay.
